# Efficacy and tolerability of sofosbuvir and daclatasvir for treatment of hepatitis C genotype 1 & 3 in patients undergoing hemodialysis- a prospective interventional clinical trial

**DOI:** 10.1186/s12882-019-1631-4

**Published:** 2019-11-28

**Authors:** Shafiq Ur Rehman Cheema, Muhammad Salman Rehman, Ghulam Hussain, Sidra Shafiq Cheema, Nooman Gilani

**Affiliations:** 1 Department of Nephrology Jinnah Hospital & Allama Iqbal Medical College, Lahore, Pakistan; 2 Department of Gastroenterology Jinnah Hospital & Allama Iqbal Medical College, Lahore, Pakistan; 3 Combined Military Hospital.L.M.C, Lahore, Pakistan

**Keywords:** Hemodialysis, Hepatitis C, Genotype 3, Daclatasvir, Sofosbuvir

## Abstract

**Background:**

There is paucity of data using direct anti-viral agents (DAA) in patients on maintenance hemodialysis (MHD) infected with HCV-genotype 1 & 3. Aim of the study was to evaluate DAA therapy in patients infected with HCV-genotype 1 & 3 on MHD.

**Methods:**

A prospective open label, parallel, non-randomized interventional trial was conducted in patients with Hepatitis-C on maintenance hemodialysis. Total of Sixty two (62) patients with hepatitis-C on maintenance hemodialysis were screened and 36 patients were enrolled and then equally allocated in 1:1 ratio to group 1 who received 400 mg daily sofosbuvir/ 60 mg daily daclatasvir and group 2 who received thrice a week 400 mg Sofosbuvir and daily 60 mg daclatasvir for 12 weeks. Patients with compensated cirrhosis received therapy for 24 weeks. Relevant data was obtained before, during and after therapy. HCV viral load was assessed at week 4, 8, at end of therapy and 12 weeks after treatment.

**Results:**

Eighteen (18) patients were allocated in each group. Three patients in group 1 withdrawn from the study after 2 weeks due to refusal to participate, while one withdrawn in group 2 due to development of adverse effect. Mean age of patients was 47.22 + 14.17 in group 1 and 53.89 + 14.11 in group 2. Genotype 3 was most common in group 1 patients, *n* = 12 (66.6%), and *n* = 11 (61.1%) in group 2. All patients in both groups achieved undetectable viral load at 12th week. As per intention to treat analysis overall 29/36 (80.55%) patients achieved SVR (group 1 = 15/18; group 2 = 14/18) and as per-protocol analysis overall 29/32 (90.62%) patients achieved SVR (group 1 = 15/15; group 2 = 14/17).

**Conclusion:**

Direct acting antiviral therapy using sofosbuvir and declatsavir is highly effective and tolerable in patients with HCV genotype 1 & 3 undergoing maintenance hemodialysis, especially when given daily.

**Trial registration:**

This trial is registered in WHO, International Clinical Trial Registry Platform, through Iranian Registry of Clinical Trials (IRCT) having IRCT ID: IRCT20170614034526N3, registered retrospectively on 2019-03-08.

## Background

The prevalence of HCV in patients on MHD ranges from 6 to 60% in different parts of the world [1]. Nosocomial transmission and spread through blood and its components are important factors that affect HCV incidence [2, 3]. Patients on dialysis are at a greater risk for progression to cirrhosis, hepatocellular carcinoma and liver-related mortality. HCV also increases the risk of serious infections in renal transplantation recipients [4]. Pegylated- interferon alone or in combination with ribavirin (RBV) have been the mainstay of treatment for HCV infection in hemodialysis patients but is associated with longer treatment duration, poor virologic response, low efficacy, lesser tolerability, high frequency of adverse effects, and requires close supportive care [5]. Direct acting antiviral (DAAs) have revolutionized the treatment of HCV infection with superior cure rates (SVR > 90%), tolerable adverse event profiles and short treatment durations but clinical data on efficacy and safety in the treatment of hemodialysis patients have been limited [6]. Direct acting antivirals including Sofosbuvir in combination with Daclatasvir, with or without ribavirin is highly effective in treating HCV infection in patients with or without cirrhosis [7–11, and]. Even immunocompromised patients can now be treated safely by interferon-free therapies, resulting in potential reduction of HCV disease [12, 13]. Certain approved options for ESRD patients includes pegylated interferon, which previously provided lower SVR rates and higher side effects [14–16, and]. Dialysis patients have been negatively impacted by HCV infection in terms of morbidity and mortality compared to non-HCV dialysis patients, this demands effective treatment option [17]. Sofosbuvir based therapy leads to high rates of SVR with few side effects [18], however use is restricted to patients who have eGFR of ≥30 ml/min per 1.73 m^2^. The active metabolite of sofosbuvir is eliminated by the kidneys and levels of sofosbuvir are substantially higher in patients with severe renal impairment [19]. Premarket testing has raised concerns for cardiovascular and hepatobiliary toxicity at higher levels of sofosbuvir dosing, but toxicity of the drug and metabolites in humans remains unknown [20]. Daclatasvir has been recommended for treatment of patients with severe renal disease, as its components are metabolized mainly by the liver. Currently, little data on the treatment of HCV in hemodialysis patients with DAAs (sofosbuvir based regimens) are available [21]. Therefore, this study was intended to assess the efficacy and tolerability of sofosbuvir based regimen in treatment of HCV in hemodialysis patients.

## Methods

### Design

Prospective, open-label, parallel, non-randomized interventional trial was conducted in dialysis Centre, The Study follows CONSORT guidelines to report the results of the trial.

### Setting & participants

Total of Sixty two (62) patients with hepatitis-C on maintenance hemodialysis were screened in dialysis centre of Jinnah Hospital Lahore, Pakistan and 36 patients were enrolled using non-probability convenient sampling procedure. The study duration was 9 months from August 1st, 2017 till April 30th 2018. As per our center policy all HCV-infected patients are isolated to a dedicated unit (HCV unit) to decrease HCV seroconversion. Strict universal precautions are used in this unit in accordance with international standards.

### Study process and interventions

**Fig. 1:**
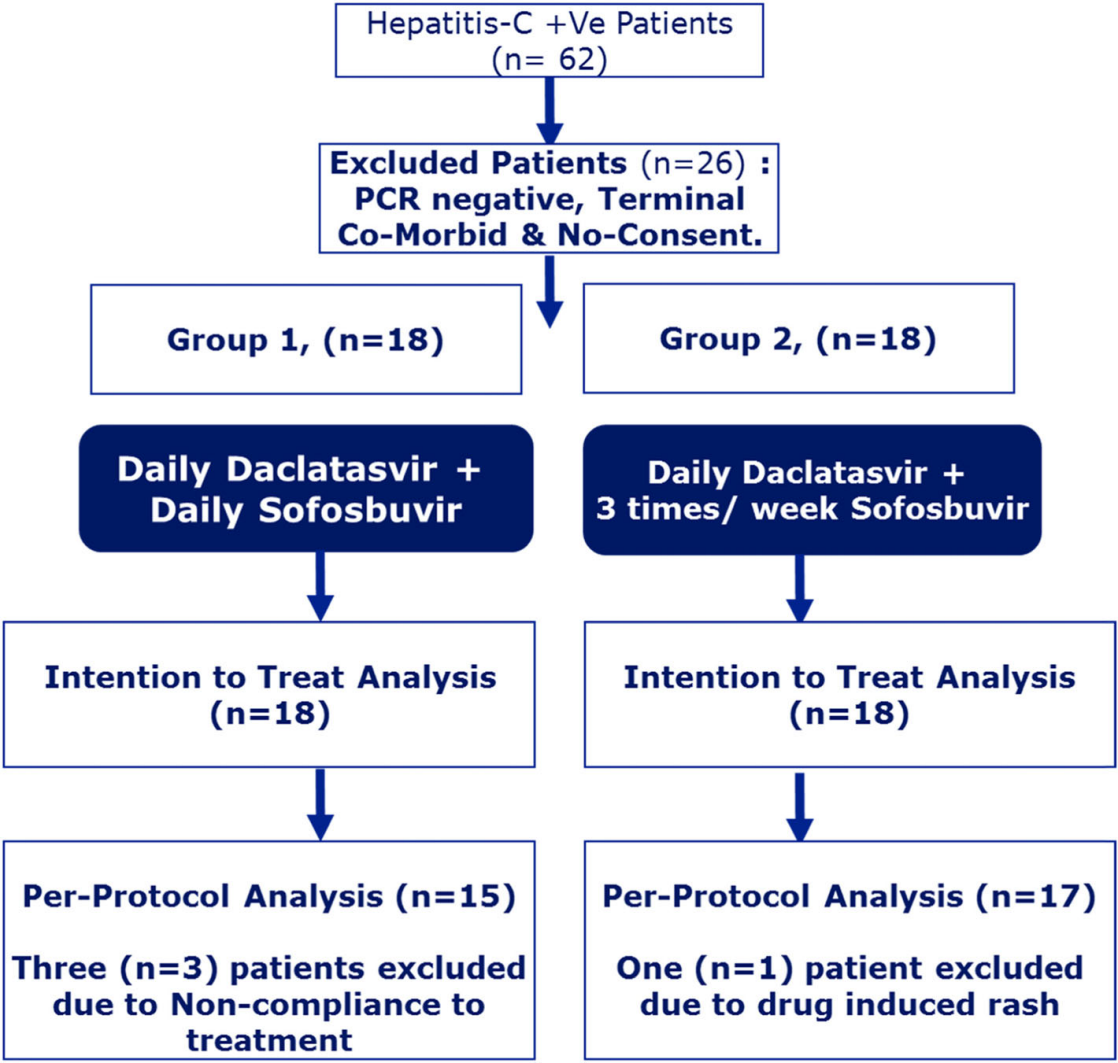
Patients Distribution in each Treatment Group

The Study was approved by Ethical Committee of Allama Iqbal medical College/ Jinnah Hospital, Lahore (39th/ ERB/ 07–2017). ESRD patients undergoing MHD in ‘HCV unit’ with detectable HCV RNA by PCR were included. Patients having co-infection with HBV, HIV, decompensated cirrhosis and terminally ill patients were excluded from the study (Fig. 1). Written informed consent was obtained from all patients. Patients were allocated in two [2] groups via convenient sampling based on treatment planned. Group 1 received daily 400 mg sofosbuvir and 60 mg daclatasvir while group 2 received three times a week sofosbuvir 400 mg and daily daclatasvir 60 mg for 12 weeks. The demographic variables and baseline investigations including complete blood count, liver function tests, HCV genotype and hepatitis C viral load were noted.

Patients were classified as having compensated cirrhosis based on clinical data, Child-Pugh score and abdominal imaging. Fibro Scan and esophagogastroduodenoscopy were performed when indicated. Patients with compensated cirrhosis were given treatment for 24 weeks. Three patients (2 with genotype 1 and one with genotype 3) in group 1 and one patient (with genotype 3) in group 2 withdrawn from the study as per protocol due to non-compliance and adverse effect respectively. However study results are reported for both intention to treat (ITT) and per-protocol (PP)  population.

### Outcomes

Quantitative HCV viral load by Real time PCR method with detectable limit of 12 IU/ml was obtained at week 4, week 8,at end of therapy, and at 12 weeks after the completion of treatment. The primary end point was achievement of SVR. An SVR was defined as undetectable viral load at 12 weeks after completion of therapy. Secondary outcome was achievement of end of treatment response (ETR) which was defined as undetectable viral load at completion of therapy.

### Statistics

All data were entered and analyzed on SPSS version 20. Frequencies and percentages were measured for the qualitative variables. Mean and standard deviations were reported for quantitative data. Independent and paired t-test were applied to check the mean difference. A *p* value of less than or equal to 0.05 was taken as significant. Primary outcomes (SVR & ETR) are analyzed and reported for Intention to treat (ITT) & per-protocol (PP) population.

## Results

This study comprised of a total of 36 patients divided into 2 equal groups depending upon the treatment. Three (*n* = 3) patients in group 1, while one (*n* = 1) patient in group 2 left treatment due to non-compliance and rash respectively. Baseline characteristics of included patients are shown in Table 1.

**Table 1:** Baseline Characteristics in each treatment group

Variables *n* = 36		Group 1 *n* = 18	Group 2 *n* = 18	*P*-Value
		Mean ± SD	Mean ± SD	
Age (Years)		47.22 ± 14.17	53.89 ± 14.11	0.17
Duration of Known Hepatitis C (Years)		4.61 ± 3.84	3.55 ± 1.92	0.31
Duration of Dialysis (Years)		4.23 ± 2.63	5.33 ± 2.79	0.23
HCV RNA PCR (log 10 IU/ml)		5.88 ± 6.0	6.16 ± 6.58	0.46
Gender	Male	*N* = 11	*N* = 11	___
	Female	*N* = 7	*N* = 7	
Genotype 1 Patients		*N* = 06	*N* = 06	___
Genotype 2 Patients		*N* = 00	*N* = 01	___
Genotype 3 Patients		*N* = 12	*N* = 11	___
Cirrhosis		*N* = 04	*N* = 06	___
Treatment Experienced		*N* = 03	*N* = 02	___
Treatment Withdrawal		*N* = 03	*N* = 01	___
Aspartate Aminotransferase (U/L)		57.06 ± 48.71	34.5 ± 25.27	0.09
Alanine Aminotransferase (U/L)		50.89 ± 44.08	40.50 ± 34.86	0.44
Hemoglobin (g/dl)		10.53 ± 1.61	11.51 ± 1.15	0.04
White Blood Cells × 10^3^/mm3		6.33 ± 1.93	6.44 ± 1.91	0.87
Platelets ×10^3^/mm3		163.27 ± 65.34	175.44 ± 40.11	0.51
Independent t-test was used to assess the significance				

After treatment all patients had significant reductions in AST and ALT values in group 1 & 2 respectively. AST & ALT values in group 1 at baseline were 57.06 ± 48.71 U/L & 50.89 ± 44.08 U/L respectively, and reduced to 20.17 ± 7.70 U/L & 20.78 ± 10.81 U/L at week 24. In group 2, baseline AST & ALT values were 34.5 ± 25.27 U/L & 40.5 ± 34.85 and at 24 week were 21.61 ± 8.23 U/L & 22.28 ± 11.92 U/L (Table 2). Three patients in group 1 had AST & ALT level greater than 100 U/L each. No significant difference was observed in complete blood indices from baseline to 24th week in both groups 1 & 2 respectively except hemoglobin level in group 2 patients (Table 2).

**Table 2:** Mean differences of Liver enzymes and hematological parameters before and after

Variables		Group*	Mean ± SDVsMean±SD	*P*-Value
Aspartate Aminotransferase U/L	Baseline VS 24th Week	1	57.06 ± 48.71Vs 20.17 ± 7.70	0.08
		2	34.5 ± 25.27 Vs 21.61 ± 8.23	0.07
Alanine Aminotransferase U/L	Baseline VS 24th Week	1	50.89 ± 44.08 Vs 20.78 ± 10.81	0.02
		2	40.5 ± 34.85 Vs 22.28 ± 11.92	0.06
Hemoglobin g/dl	Baseline VS 24th Week	1	10.53 ± 1.61 Vs 10.31 ± 1.69	0.70
		2	11.51 ± 1.14 Vs 10.02 ± 1.712	0.002
White Blood Cells ×10^3^/mm3	Baseline VS 24th Week	1	6.33 ± 1.94 Vs 5.78 ± 1.16	0.37
		2	6.44 ± 1.91 Vs 6.61 ± 1.64	0.78
Platelets ×10^3^/mm3	Baseline VS 24th Week	1	163.27 ± 65.90Vs 172.94 ± 62.74	0.61
		2	175.44 ± 40.11 Vs 184.89 ± 53.82	0.58
Paired t-test was used to assess the significance				

Treatment in Group 1 & Group 2 Patients (Per-Protocol Population)

* Patient Treatment Groups:

Group 1: Daily Daclatasvir + Sofosbuvir,

Group 2: Daily Daclatasvir + 3 times/week Sofosbuvir

As per ITT analysis Rapid Virological Response; RVR (Undetectable HCV-RNA after 4 weeks of therapy) was achieved in 14/18 patients (77.7%) in group 1 compared to 15/18 patients (83.3%) in group 2. End of Treatment response; ETR (undetectable HCV-RNA at completion of therapy) was achieved in 15/18 patients in group 1 (83.3%) and 17/18 patients in  group 2 (94.4%) respectively. SVR (undetectable HCV-RNA 12 weeks after end of treatment) was achieved in 15/18 patients (83.3%) in group 1 compared to 14/18 patients (77.7%) in group 2 (Table 3).

**Table 3:** Viral load, RVR, ETR &SVR in Group 1 and Group 2 (Intention to Treat Analysis)

	Viral load detectable			
	Group 1(*n* = 18)		Group 2 (*n* = 18)	
	No	Yes	No	Yes
	N (%)	N (%)	N (%)	N (%)
4th Week (RVR)	14 (77.7)	1 (6.6)	15 (83.3)	2 (11.1)
8th Week	15 (83.3)	(0)	17 (94.4)	0 (0)
12th Week (ETR)	15 (83.3)	(0)	17 (94.4)	0 (0)
24th Week (SVR)	15 (83.3)	(0)	14 (77.7)	3 (16.6)

**Fig. 2:**
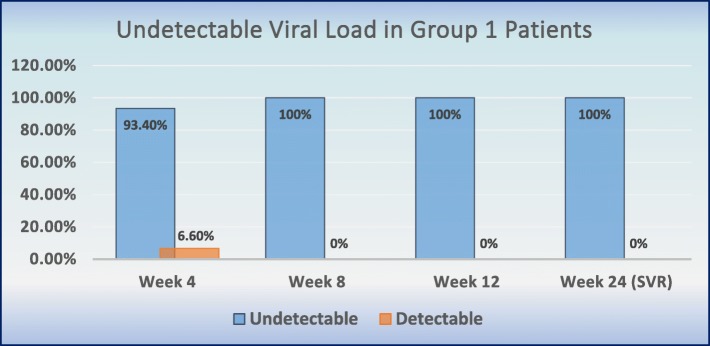
Undetectable Viral load in Group 1 Patients (Per-Protocol Population)

**Fig. 3:**
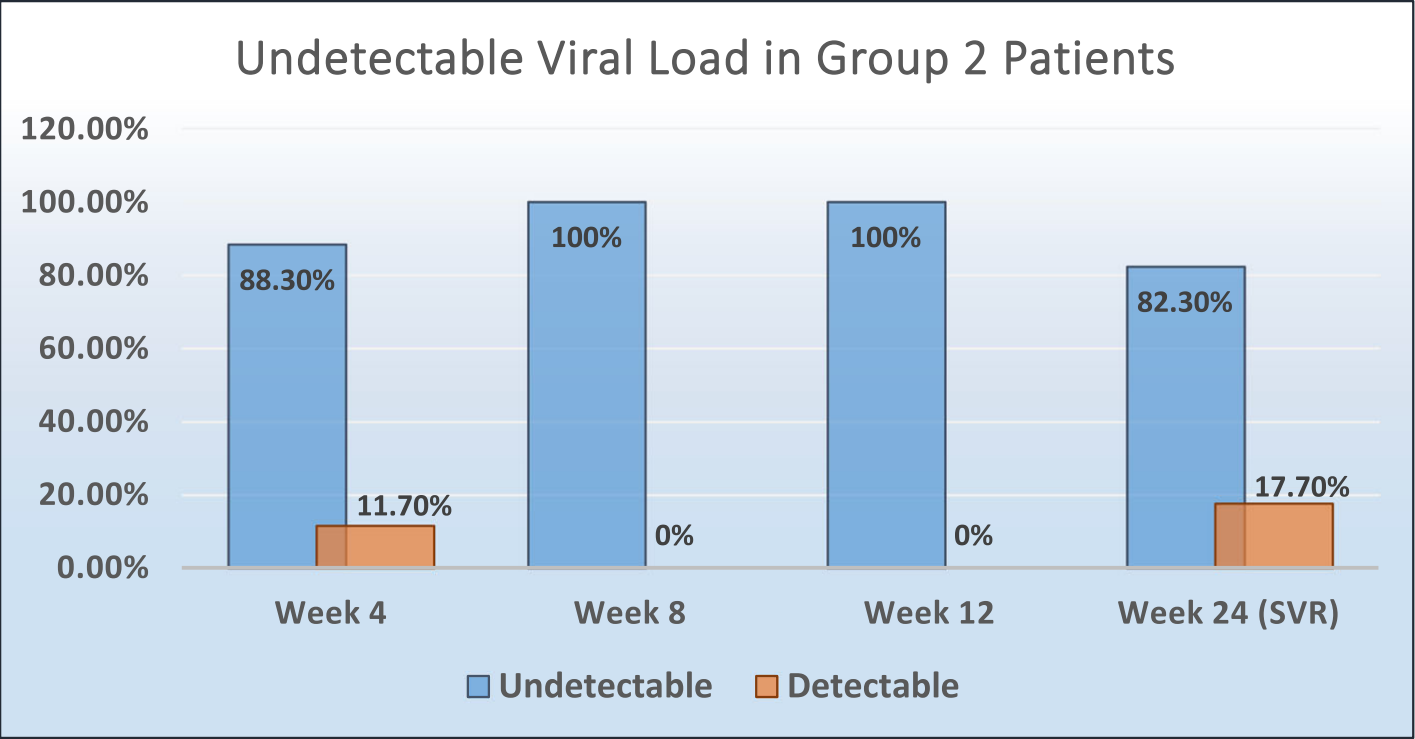
Undetectable Viral load in Group 2 Patients (Per-Protocol Population)

As per PP population, RVR was achieved in 14/15 patients (93.4%) in group 1 compared to 15/17 patients (88.3%) in group 2. ETR was achieved in all patients in group 1 (15/15; 100%) and group 2 (17/17; 100%) respectively. SVR was achieved in 15/15 patients (100%) in group 1 compared to 14/17 patients (82.35%) in group 2 (Table 4, Figs. 2 and 3). One patient with cirrhosis in group 2 did not achieve RVR but all cirrhotic patients in both groups achieved ETR and SVR. Three patients in group 2 who did not achieved SVR were all infected by genotype 1 and two of them had previously received treatment with interferon (Table 5).

**Table 4:** Viral load, RVR, ETR &SVR in Group 1 and Group 2 (Per Protocol-Analysis)

	Viral load detectable			
	Group 1(*n* = 15)		Group 2 (*n* = 17)	
	No	Yes	No	Yes
	N (%)	N (%)	N (%)	N (%)
4th Week (RVR)	14 (93.4)	1 (6.6)	15 (88.3)	2 (11.7)
8th Week	15 (100)	0 (0)	17 (100)	0 (0)
12th Week (ETR)	15 (100)	0 (0)	17 (100)	0 (0)
24th Week (SVR)	15 (100)	0 (0)	14 (82.3)	3 (17.7)

**Table 5:** Group 2 Patients not achieving SVR

Gender	Cirrhosis	Previous Treatment	Baseline PCR (log 10 IU/ml)	ETR	SVR12 PCR (log 10 IU/ml)	Genotype
Male	No	Yes	5.86	Not Detected	5.51	1
Female	No	No	5.60	Not Detected	4.11	1
Male	No	Yes	5.86	Not Detected	5.50	1

## Discussion

As per published data, HCV is endemic in Pakistan and around 6.8% of Pakistani population might be infected with HCV which is almost a 40% increase in HCV sero-prevalence in recent years [22]. It appears that SOF based regimens are currently the choice to treat hepatitis-C and once-daily oral sofosbuvir plus daclatasvir is associated with high rates of SVR among patients infected with HCV genotype 1, 2, or 3 [11]. Patients who have advanced CKD are less likely to receive treatment for HCV despite of safety and efficacy data [23]. There are various published studies that report the efficacy & safety of DAAs in Hepatitis-C patients with advanced chronic kidney disease in Japan; daclatasvir & asunaprevir in genotype 1 patients [24], glecaprevir & pibrentasvir combination in genotype 1, 2 & 3 patients [25], ombitasvir, paritaprevir & ritonavir in genotype 1 b dialysis patients [26]. Certain newer DAAs as mentioned earlier are not yet available in Pakistan that could be safely used in patients with advanced CKD [22]. So considering the situation, this study is by far the largest from Pakistan reporting that SOF based therapy can be safely and effectively used to treat HCV in patients undergoing MHD.

In recently published meta-analysis using DAAs in MHD, the SVR in all these studies ranged from 66.7 to 98.3% but in a subgroup with SOF-based therapy, SVR was 89.4% [27]. This is consistent with our study results showing overall SVR of 80.55% in intention to treat population and SVR of 90.62% in per-protocol population. In one of the meta-analysis nineteen patients in 2 studies were treated with half dose of SOF and 4 of them failed to achieve SVR. Similarly, in our study, all patients who did not achieve SVR were from the group in which 3 times per week SOF was used (SVR; 77.7% in ITT population & SVR; 82.3% in PP population) indicating the dose to be an important variable to achieve SVR. In most studies SOF was well tolerated like in our study, where only one patient left the study due to rash which improved after DAAs were stopped. In the meta-analysis, genotype 1 was the most common, unlike in our study where genotype 3 was most prevalent. In our study cohort, we had 12 patients (33.3%) with genotype 1, 01 patient (2.7%) with genotype 2 and 23 patients (63.8%) with genotype 3, which remains to be the most prevalent genotype in Pakistan [22].

In a recent study from India [28], that included treatment-naïve haemodialysis, HCV infected patients. Most patients had genotype 1 (64.5%), followed by genotype 3(29%). Patients were treated with different frequency of drug usage, like daily SOF/Ribavirin, every other day SOF /ribavirin, daily SOF/daclatasvir and every other day SOF/daclatasvir for 12 weeks. 95.2% achieved SVR. There was no impact of genotype on SVR. Treatment with daily daclatasvir and daily sofosbuvir yielded a 93.3% (14/15) SVR, compared to 100% (6/6) SVR with daily daclatasvir and alternate day sofosbuvir. Our study reported a 100% SVR in daily SOF/daclatasvir group and 82.35% in thrice weeklySOF/daclatasvir group. The characteristics of group 2 patients who achieved ETR but not SVR12 are shown in Table 2.

In our study population most common genotype was 3 (63.8% of study population) and all patients with genotype 3 achieved SVR with either daily sofosbuvir or thrice/ week Sofosbuvir based regimen. This is also comparable to Agarwal et al. study [28] in which 29% of hemodialysis patients treated for hepatitis-C had genotype 3 and overall 95.9% of patients had achieved SVR.

In one prospective study [29], two dosing regimen were compared. One group received daily sofosbuvir (*N* = 7) and other group received three times a week sofosbuvir (*N* = 5) along with simeprevir, daclastavir, ledipavir or ribavirin. Both groups showed higher SOF-007 plasma concentrations. Sofosbuvir or its inactive metabolite was not accumulated with either regimen, irrespective of hemodialysis sessions or treatment course. Study participants experience no serious adverse event. SVR was achieved in 10 out of 12 patients (83%). Two relapses occurred with 3 times a week regimen and none with the daily regimen. In our study overall SVR was 90.62% and all 3 relapses occurred only in thrice weekly regimen.

Gane et al. [30] presented results of 10 patients with severe renal impairment (9 infected with HCV genotype 1, and one with genotype 3) receiving 200 mg daily sofosbuvir, combined with 200 mg daily RBV. Although, the dose was safe, the regimen was not efficacious and resulted in an SVR of only 40%.This regimen resulted in 6 relapses in HCV genotype 1 infected patients who had previously achieved SVR. This emphasizes the fact that full dose of SOF is an important variable to achieve SVR.

Similarly, Bhamidimarri et al. [31] evaluated *2* different schedules in 15 patients with severe renal impairment (*n* = 3) or requiring hemodialysis (*n* = 12). Eleven patients received sofosbuvir, 200 mg daily, and 4 patients received sofosbuvir 400 mg three times weekly, all with simeprevir at a standard dose. Results demonstrated an overall SVR of 87% with no major toxicity observed in either group. Two relapses occurred, one in each group.

Our study like few others have shown that even in patients with ESRD on MHD who usually have eGFR of less than 10 ml/min/1.73m^2^, SOF based regimen are not only effective but tolerable as well. Given higher SVR with daily SOF based regimen at full dosages, this approach appears preferred in this patient population. Further studies on a larger scale are pivotal especially in developing countries where there is higher prevalence of HCV in MHD patients.

## Conclusion

Hepatitis C virus infection is endemic in Pakistan like some other developing courtiers and its burden is expected to increase in patients with hemodialysis in coming years owing mainly to widespread use of unsafe medical procedures. Daily Full dose sofosbuvir in combination with daclatasvir is very effective and appears tolerable in ESRD patients with genotype 1 & 3 undergoing maintenance hemodialysis. Decreasing the dose or frequency of SOF may lead to decreased SVR or higher relapses. Larger scale studies are warranted since sofosbuvir is backbone of direct acting antivirals in developing world not only because of its efficacy but due to its low cost as well.

## Data Availability

The datasets used and/or analyzed during the current study available from the corresponding author on reasonable request.

## Abbreviations

DAA: Direct acting anti-viral
ETR: End of Treatment Response
HCV: Hepatitis-C Virus
RVR: Rapid Virological Response
SVR: Sustained Virological Response
